# The Gln27Glu Polymorphism in β2-Adrenergic receptor gene is linked to hypertriglyceridemia, hyperinsulinemia and hyperleptinemia in Saudis

**DOI:** 10.1186/1476-511X-9-90

**Published:** 2010-08-25

**Authors:** Maha H Daghestani, Arjumand Warsy, Mazin H Daghestani, Ali N Al-odaib, Abdelmoneim Eldali, Nadia A Al-Eisa, Sabah Al-zhrani

**Affiliations:** 1 Department of Zoology, Center for Scientific and Medical Female Colleges, King Saud University, P.O. Box 22455, Riyadh, Saudi Arabia; 2 Department of Biochemistry, Center for Scientific and Medical Female Colleges, King Saud University, P.O. Box 22452, Riyadh, Saudi Arabia; 3 Department of Obstetrics & Gynecology, Umm-Al-Qura University, P.O.Box 424, 21955, Makkah, Saudi Arabia; 4 Department of Genetics, King Faisal Specialist Hospital and Research Center P.O. Box 3354, Riyadh 11211, Saudi Arabia; 5 Biostatistics, Epidemiology & Scientific Computing (MBC - 03), King Faisal Specialist Hospital and Research Center, P. O. Box 3354, Riyadh 11211, Saudi Arabia

## Abstract

**Background:**

β2-adrenoceptor (β2AR) gene polymorphism glutamine 27 glutamic acid (Gln27Glu) and Arg16Gly were reported to have an association with obesity and obesity related disorders in some population. We evaluated Gln27Glu polymorphism in the β2AR gene in obese Saudi populations to investigate the association of β2AR gene with obesity and other related metabolic parameters.

**Design:**

We studied possible association of Gln27Glu in β2AR gene with body mass index (BMI), anthropometric measurements and other metabolic parameters. The β2AR gene polymorphism (Gln27Glu) was identified by sequencing PCR products representing locus of interest. Based on BMI, the subjects were divided into three groups, normal weight, overweight and obese. The genotype and allele frequency were calculated separately for each group.

**Results:**

The allelic frequency of Glu27 did not differ amongst the three groups, though the Glu27 homozygote (Glu/Glu) were more in obese subjects and had higher concentration of triglyceride, leptin and insulin compared to in the Gln27 heterozygotes and Gln/Gln homozygotes.

**Conclusions:**

In this study we were able to provide evidence on the influence of Gln27Glu genetic variant of β2AR gene on lipid phenotypes, insulin and leptin levels in the Saudi populations.

## Background

Obesity is serious health problem among men and women. It is a multifactorial phenotype determined by genetic and non genetic factors including environmental and behavioral characteristics [1]. Extensive studies have been conducted in different populations to identify the loci contributing to obesity development. In the process, a complex picture has emerged where inter-population differences in the susceptibility genes are observed. It has been observed that catecholamines act through both β- and α adrenergic receptor which mediate through different receptor pathways [2]. Genes involved in the regulation of catecholamine function may be of particular importance for human obesity, since catecholamine via β2-3 adrenergic receptors or adrenoceptors, regulate body fat accumulation and play a central role in the regulation of energy expenditure [3]. This regulation is in part affected by the stimulation of lipid mobilization through lipolysis in fat cells, which has been associated with obesity [4,5]. β2AR are expressed in many cell types throughout the body and play a crucial role in the regulation of the cardiac, pulmonary, vascular, endocrine and central nervous system. Several functional polymorphisms occur within the coding region of this gene. The Gln27Glu polymorphism in the β2AR gene has been considered important factor contributing to obesity [6]. Among these, a common mutation results is the substitution of an amino acid in the extracellular domain of the receptor, Gln27Glu [7]. This appears to modify the extracellular part of the β2AR with possible functional modification.

Recent studies on some ethnic groups have shown that this polymorphism is associated with obesity, insulin resistance and dyslipidemia [8-10]. However, reports from other populations contradict these results [11-13]. In some studies, gender differences in relation to the polymorphism are reported [14-16]

In an attempt to elucidate the influence of the β2AR polymorphisms on obesity development, we investigated the association of Gln27Glu gene in obesity and its influence on other metabolic profiles.

## Materials and methods

### Subjects

This study included 329 unrelated Saudi men (n = 109, 33.1%) and women (n = 220, 66.9%) aged 18 to 36 years, randomly chosen from a nationwide population. All the study subjects gave their written consent to participate in the study after being informed of its nature. The study was approved by local ethical committee of the university. They agreed to have venous blood drawn after an overnight fasting. For laboratory studies, 20 ml of blood was obtained in the morning following an overnight (12 hour) fast. Blood samples were collected in EDTA-coated tubes and immediately centrifuged at room temperature. The buffy coat and the plasma were stored at -20°C until required for analysis.

### Anthropometric Measurements

For each individual, anthropometric measurements were made using standardized techniques. These included measurement of height, weight, waist and hip circumference and these were used to calculate BMI (kg/m^2^), and waist-to-hip ratio (WHR).

### Biochemical Parameters

Plasma glucose level was estimated from the fresh samples by glucose-oxidase method adapted to an autoanalyzer (Hitachi 704, Boehringer Mannheim, GmBH, Germany). Frozen plasma samples were thawed at room temperature and used for the analysis of the other biochemical parameters. The estimation of total cholesterol, triglycerides, high density lipoprotein (HDL)-cholesterol and low-density lipoprotein (LDL)-cholesterol were carried out by enzymatic methods using commercial kits (Boehringer Mannheim). Plasma insulin and leptin concentrations were determined by radioimmunoassay (RIA; Human Insulin Specific RIA kit and Human Leptin RIA Kit, respectively, Linco Research, St Louis, MO). All analyses were conducted in duplicates. Quality control was maintained within and between the batches.

### Genotyping of β2AR Polymorphism

Genomic DNA was extracted from peripheral blood leucocytes using commercially available Gentra Systems Kit (Minneapolis, MN, cat # D5500). The concentration and purity of the DNA were estimated prior to analysis and DNA was used for the following studies:

The DNA segment representing codon 27 of the β2AR gene was amplified by polymerase chain reaction (PCR) using forward (5'-AAGCTGAGTGTGCAGGACGA-3') and reverse primers (5'-AGACGCTCGAACTTGGCAAT-3'). The PCR conditions consisted of an initial denaturation step at 95°C for 15 minutes, followed by 34 cycles of denaturation at 95°C for 1 minute, annealing at 64°C for 1 minute, and extension at 72°C for 1 minute, with a final extension of 10 minutes at 72°C. A PCR product of 353 bp was obtained. Nucleotide sequencing was carried out using the ABI Big Dye Terminator protocol on ABI 3100 Avant Genetic Analyzer.

### Statistical analysis

The subjects were grouped as normal weight (BMI < 25 Kg/m^2^), overweight (BMI 25-29.9 Kg/m^2^) and obese (BMI > 30 Kg/m^2^), based on their BMI conditions. The analysis was done using the Stat View program for Windows (version 8.0; SAS) and the SPSS (Statistical Package for Social Sciences; Version 13).

The data are presented as mean ± SEM for the males and females separately. The comparisons of the results of the overweight and obese subjects with the results of the matched control were carried out using the independent Students T-test and ANOVA test. The distributions of the age, BMI, anthropometric measurements, lipid profile, fasting serum insulin, leptin and glucose values of the study subjects according to their β2AR gene were compared by the U Mann-Whitney test. Multivariable logistic regression was used to study the effect of the β2AR genotype on BMI. Genotype and allele frequency of β2AR alleles were calculated separately for the normal weight, overweight and obese individuals and compared using chi square analysis. Frequency distribution analysis was performed with chi square test. Relative risk was estimated by the odds ratios (ORs) and their 95% confidence intervals (CIs). A probability value p ≤ 0.05 was considered statistically significant.

## Results and discussion

Based on the BMI value, there were 35, 28, 46 males and 80, 40, 100 females in the normal weight, overweight and obese groups, respectively. The demographic characteristics of the male and female study subjects are presented in Table 1. All the three groups matched in their age and no significant differences were observed amongst the groups. All other demographic parameters were significantly higher in overweight and obese subjects compared with normal weight controls (p < 0.0001). The biochemical parameters in the three groups of males and females are presented in Table 2. All parameters were significantly elevated in the overweight and obese subjects, except HDL-cholesterol, which was significantly lower compared to the normal weight group.

**Table 1:** Mean comparisons of anthropometric characteristics of the overweight and obese groups with the normal control group.

VARIABLES	Control Lean (n = 115) mean ± SE	Overweight (n = 68) mean ± SE	Obese (n = 146) mean ± SE	P value
Age (yr)				

M	25.9 ± 0.7	27.3 ± 0.67	26.7 ± 0.73	0. 056
		
F	24.5 ± 0.5	24.4 ± 0.7	26.7 ± 0.54	
	
T	24.90 ± 0.44	25.56 ± 0.54	26.70 ± 0.43	

BMI (kg/m^2^)				

M	21.27 ± 0.34	26.4 ± 0.27	32.9 ± 0.8	0.0001
		
F	21.4 ± 0.20	27.3 ± 0.2	35.8 ± 0.6	
	
T	21.33 ± 0.189	26.93 ± 0.170	34.89 ± 0.50	

Waist (cm)				

M	74.2 ± 1.53	86.1 ± 1.4	110.5 ± 2.2	0.0001
		
F	67.3 ± 0.9	84.4 ± 1.23	100.6 ± 1.38	
	
T	70.82 ± 0.79	85.13 ± 0.93	103.72 ± 1.23	

Hip (cm)				

M	97.6 ± 1.2	100.8 ± 1.17	122.2 ± 2.0	0.0001
		
F	95.5 ± 0.8	102.2 ± 1.1	118.5 ± 1.4	
	
T	96.13 ± 0.68	102.22 ± 0.821	119.68 ± 1.148	

W/H Ratio				

M	0.76 ± 0.02	0.86 ± 0.01	0.90 ± 0.01	0.0001
		
F	0.72 ± 0.01	0.82 ± 0.02	0.85 ± 0.007	
		
T	0.73 ± 0.006	0.84 ± 0.006	0.87 ± 0.006	

NOTE: Values are mean ± SEM. Abbreviations: BMI, body mass index; W/H, waist hip ratio**; **M= Male; F= Female; T= Total.

**Table 2:** Comparison of the biochemical parameters of the overweight and obese groups with the normal control group

VARIABLES	Control Lean (n = 115) mean ± SE	Overweight (n = 68) mean ± SE	Obese (n = 146) mean ± SE	P value
Cholesterol (mmol/L)				

M	3.31 ± 0.07	4.4 ± 0.15	4.1 ± 0.11	0.0001
	
F	3.5 ± 0.05	4.05 ± 0.12	4.5 ± 0.09	
	
T	3.43 ± 0.043	4.194 ± 0.096	4.4 ± 0.074	

Triglyceride (mmol/L)				

M	0.79 ± 0.05	1.15 ± 0.08	1.43 ± 0.07	0.0001
	
F	0.72 ± 0.05	1.03 ± 0.06	1.24 ± 0.04	
	
T	0.742 ± 0.024	1.082 ± 0.05	1.302 ± 0.04	

HDL-cholesterol (mmol/L)				

M	1.28 ± 0.06	1.21 ± 0.07	1.08 ± 0.04	0.0001
	
F	1.34 ± 0.04	1.17 ± 0.05	1.06 ± 0.03	
	
T	1.32 ± 0.033	1.19 ± 0.043	1.07 ± 0.024	

LDL -cholesterol (mmol/L)				

M	1.58 ± 0.09	2.25 ± 0.15	2.4 ± 0.11	0.0001
	
F	1.53 ± 0.06	1.94 ± 0.11	2. 6 ± 0.07	
	
T	1.55 ± 0.05	2.07 ± 0.89	2.56 ± 0.059	

Leptin (ng/ml)				

M	3.10 ± 0.17	9.91 ± 0.38	17.6 ± 0.86	0.0001
	
F	12.2 ± 0.4	22.84 ± 0.97	44.6 ± 1.85	
	
T	9.42 ± 0.48	17.51 ± 0.97	36.13 ± 1.67	

Fasting Insulin (pmol/L)				

M	62.6 ± 4.23	67.07 ± 5.27	111.9 ± 5.6	0.0001
	
F	54.85 ± 2.33	75.84 ± 6.26	103.3 ± 3.64	
	
T	57.205 ± 2.089	72.23 ± 4.28	106.03 ± 3.066	

Fasting Glucose (mmol/L)				

M	4.84 ± 0.07	5.16 ± 0.05	5.06 ± 0.09	0.0001
	
F	4.65 ± 0.05	4.97 ± 0.08	5.06 ± 0.06	
	
T	4.71 ± 0.041	5.051 ± 0.057	5.06 ± 0.05	

NOTE: Values are mean ± SEM. Abbreviations: BMI, body mass index; W/H, waist hip ratio**; **M= Male; F= Female; T= Total.

The genotype and allele frequency of the Gln27Glu polymorphism was calculated in the male and females and the results are presented in Table 3. The overweight and obese subjects were separated from the normal weight group and the Gln27Glu genotype and allele frequency was calculated separately in each group. The allelic frequency was not elevated significantly in the overweight and obese female and male groups. However, the Glu27 homozygote had significant greater BMI, waist and hip circumference and significant higher concentration of triglyceride, leptin and insulin compared to the Gln27 homozygote (Table 4).

**Table 3:** Distribution of the genotypes, alleles frequencies and odd ratio of the β2 Gln27Glu polymorphism in overweight and control subjects.

*Genotype*	*Sex*	*Control* *M:35* *F: 80* *T = 115*	*Overweight* *M:28* *F: 40* *T = 68*	*Obese* *M:46* *F: 100 T = 146*	*χ2* *Odds ratio(95% CI)*	*P- value*
***Genotype Frequency***						

**Gln\Gln**	M	24 (68.57)	17 (60.7)	27 (58.67)	**χ2** M= 1.19 F= 2.23	***P value*** M = 0.880
			
	F	51 (63.75)	26 (65)	60 (60)		
			
	T	75(65.2)	43(63.2)	87(59.6)		
		
**Gln\Glu**	M	5 (14.28)	5 (17.58)	7 (15.21)	***Odds ratio (95% CI) Control vs OW*** 1.1 (0.5, 2.39)	F = 0.693
			
	F	13 (16.25)	6 (15)	12 (12)		
			
	T	18(15.7)	11(16.2)	19(13.0)		
		
**Glu\Glu**	M	6 (17.12)	6 (21.42)	12 (26)	***Odds ratio (95% CI) Control vs Obese*** 1.567(0.85,2.87)	***P value for Odds ratio in normal vs overweight and normal vs obese:*** P > 0.05
			
	F	16 (20)	8 (20)	28 (28)		
			
	T	22(19.1)	14(20.6)	40(27.4)		

***Allele Frequency***						

**Allele Gln27**	T	73.0%	71.3%	66.1%	***Odds ratio (95% CI) Control vs OW*** 0.95 (0.71, 1.27)	P = 0.70
		
**Allele Glu27**	T	27.0%	28.7%	33.9%	***Odds ratio (95% CI) Control vs Obese*** 0.87 (0.74, 1.2)	P = 0.09

NOTE: M= Male; F= Female; T= Total; OW= Overweight; CI: Confidence interval

**Table 4:** Phenotypic Characteristics of the Subjects According to β2AR Polymorphism at Codon 27

Variables	Gln\Gln (n = 205) *mean ± SE*	Gln\Glu (n = 48) *mean ± SE*	Glu\Glu (n = 76) *mean ± SE*	*P-value*
BMI (kg/m^2^)	27.917 ± .508	27.880 ± .96	30.483 ± .908	0.03

Waist(cm)	86.51 ± 1.213	88.10 ± 2.799	93.59 ± 2.390	0.02

Hip(cm)	106.15 ± 1.016	108.38 ± 2.350	112.07 ± 1.767	0.014

W/H ratio	.8112 ± .006	.8098 ± .014	.8300 ± .011	0.3

Cholesterol (mmol/L)	3.996 ± .06	3.944 ± 104	4.117 ± .105	0.48

Triglyceride (mmol/L)	.980 ± .030	1.088 ± .064	1.260 ± .057	0.0001

HDL-cholesterol (mmol/L)	1.2093 ± .0235	1.1683 ± .0525	1.1204 ± .0375	0.142

LDL-cholesterol (mmol/L)	2.074 ± .0562	2.11 ± .11	2.179 ± .09	0.62

Leptin (ng/ml)	21.39 ± 1.190	21.99 ± 2.62	27.74 ± 2.549	0.04

Fasting Insulin (pmol/L)	78.525 ± 2.758	79.779 ± 4.677	92.683 ± 4.632	0.02

Fasting Glucose (mmol/L)	4.911 ± .038	5.000 ± 095	4.961 ± .0438	0.53

NOTE: Values are mean ± SEM. Abbreviations: BMI, body mass index; W/H, waist hip ratio.

The main findings of the present study show that the male and female overweight and obese subjects do not differ in the genotype and allele frequencies of Glu27, however, the subjects who are Glu27 homozygote have greater BMI, waist and hip circumference, but not waist-to-hip ratios (abdominal obesity). These are associated with higher levels of triglyceride, serum leptin and insulin levels. There were no difference in the alleles frequency amongst overweight, obese and the control lean groups. These observations demonstrate that the Glu27 genotype of the β2AR gene is related to some obesity phenotypes and higher triglyceride, insulin and leptin levels. Our results are in agreement with those of many investigators who reported that the Glu27Gln genotype of the β2AR gene has a well established association with obesity [14]. Subjects who are Glu27 homozygotes have excess body fat and increased fat cell size compared with Gln homozygotes in a white population and also suffers from abdominal obesity and insulin resistance [15]. Similarly in another study Kunnas et al., observed significantly higher amount of visceral fat in women with Glu allele compare to Gln/Gln homozygote [17]. Masuo et al., have reported that the mutations in the *β2AR *gene are associated with increased insulin resistance, adiposity, and blood pressure accompanied by higher plasma norepinephrine and leptin levels[16]. Ishiyama et al., have suggested an association of polymorphisms in the β2AR receptor gene with obesity, hypertriglyceridemia, and diabetes mellitus in Japanese subjects [18]. They observed that the Gln27Glu heterozygotes were twice as common in obese subjects, and that the frequency of the Glu27 allele was also higher in patients with type 2 diabetes mellitus than in nondiabetic subjects [18]. Another Japanese study reported that the Gln27Glu β2AR receptor variant was associated with obesity in males [19]. Ukkola et al., found that gene-to-gene interactions among the α_2_-, β_2_-, and β-adrenergic receptor genes contributed to the phenotypic variability in abdominal obesity and plasma lipid and lipoprotein in a family study in Québec [20]. Though, these and other studies show an association between the Gln27Glu, however, there is considerable debate on this association, because there have been several studies that have failed to show the significant association between β2AR gene polymorphism and obesity [11-13]. Hayakawa et al., reported that Gln27Glu and Arg16Gly polymorphisms of the β2AR gene are not a major contributing factor to obesity, blood pressure, serum lipid levels, uric acid, or free fatty acid levels in 210 Japanese men [11]. Oberkofler et al., reported that Gln27Glu polymorphisms in the β2AR gene is not a major factor contributing to morbid obesity in Austrian women [13]. Kim et al., found that the Glu27Glu and Arg16Gly polymorphisms of the β2AR gene are not major factor contributing towards obesity in Korean subjects [21].

Furthermore, a few studies report gender-specific association between β2AR polymorphism and obesity. Hellstrom et al., suggested that a positive association between obesity and the Glu27 genetic variant in β2AR exists in females, whereas there is a negative correlation between Glu27 and obesity in Swedish male subjects [15]. Although the reasons for this discrepancy among studies are unclear, they may be due to the difference in the degree of obesity among study subjects or may be due to genetic variations between ethnic groups. However, further studies in larger populations are required to verify these results.

Our findings provide strong evidence on the influence of the Gln27Glu genetic variants on lipid phenotypes, insulin and leptin levels in overweight/obese Saudi subjects.

## Competing interests

The authors declare that they have no competing interests.

## Authors' contributions

MHD designed the experiment, analyzed the data and wrote the manuscript. AW helped in writing the draft of the manuscript and in statistical analysis. ANO consultation and result interpretation. MHD arranged the subjects/samples of the study. NAA helped in result interpretation. AE performed all the statistical analysis and SZ performed biochemical analysis.

All authors read and approved the final manuscript.
